# Natural variability of trace-amine associated receptors in wild meerkats

**DOI:** 10.1186/s12983-025-00590-2

**Published:** 2025-11-27

**Authors:** Johannes Fähnle, Kerstin Wilhelm, Benedikt Wiese, Marta Manser, Tim Clutton-Brock, Simone Sommer, Nadine Müller-Klein

**Affiliations:** 1 Institute for Evolutionary Ecology and Conservation Genomics, Ulm, Germany; 2 Department of Evolutionary Biology and Environmental Studies, University of Zurich, Zurich, Switzerland; 3 Mammal Research Institute, University of Pretoria, Pretoria, South Africa; 4 Kalahari Research Trust, Kuruman River Reserve, Northern Cape South Africa; 5 Large Animal Research Group, Department of Zoology, University of Cambridge, Cambridge, UK

**Keywords:** Meerkat (Suricata suricatta), olfaction, Trace-amine associated receptor, high throughput sequencing

## Abstract

**Background:**

The study of olfaction as a sensory modality has been relatively neglected in comparison to other sensory cues, particularly in wildlife research. Furthermore, the genetic basis of olfaction remains poorly understood in non-model species. Recently, receptors belonging to trace amine-associated receptor (TAAR) family have been identified, and they exhibit considerable natural diversity and copy number variations across a range of species. As such, they represent a promising avenue for exploring olfactory processes, particularly in conjunction with more established olfactory receptors. In meerkats (*Suricata suricatta*), olfaction plays a significant role in foraging, social communication and predator defence. However, no prior research has investigated the genetics of meerkat olfaction. In this study, we leveraged the extensive longitudinal dataset from the Kalahari Meerkat Project, using samples from 398 individuals alive between 1996 and 2023, to develop a high-throughput sequencing approach for assessing TAAR diversity in wild meerkats.

**Results:**

A total of nine TAAR-loci were identified in meerkats, with both copy number variations and allelic polymorphisms observed for TAAR6 and TAAR8. Two distinct paralogues of TAAR6 and eleven distinct amino acid alleles across these paralogues were identified. Additionally, three paralogues of TAAR8, containing 14 distinct amino acid alleles, were discovered. Within each paralogue of both TAAR loci, a single allele is present in almost all individuals, while additional alleles show a markedly higher degree of variability in frequency. A similar pattern emerges in the relative abundance of TAAR alleles throughout the course of the study, which spanned more than 20 years. In line with the high prevalence of specific alleles and the considerable number of synonymous nucleotide exchanges, we found evidence for multiple sites under purifying selection in TAAR6 and TAAR8.

**Conclusion:**

This is the first study to examine TAAR diversity in a cooperative breeder. Investigating the genetic basis of olfaction and inter-individual natural variation in TAAR diversity has the potential to expand the toolbox for integrative zoological research. Such insights could help elucidate the genetic underpinnings of behaviour, such as social communication, mate choice, and life-history strategies all in relation to TAAR diversity.

**Supplementary Information:**

The online version contains supplementary material available at 10.1186/s12983-025-00590-2.

## Background

Chemosensing, specifically the detection of olfactory cues, has historically received less attention than other sensory modalities in mammals. Deciphering the genetic basis of olfaction in mammals is extremely challenging due to the huge diversity among olfactory receptors (ORs), with several hundreds or even over 1000 classical ORs reported for a range of species (reviewed in [1]). In addition to the classical ORs, a second class of receptors has emerged as a probable, albeit less diverse, receptor family involved in olfactory sensing over the past three decades, namely the trace-amine associated receptors (TAARs) [2, 3]. Classical ORs and TAARs share a number of important characteristics: Both belong to the same superfamily, the G protein-coupled receptors, and as such share the structure of seven α-helical transmembrane domains and their activation of the intracellularly bound G protein upon ligand binding, facilitating intracellular signal transduction cascades [4]. Both ORs and TAARs also show remarkably similar expression patterns within the olfactory epithelium [5], where each olfactory neuron expresses one TAAR or one OR gene [3, 6]. While the extremely high variability of ORs makes their study challenging, the smaller TAAR repertoire, coupled with strong links to olfaction, makes them a highly intriguing target for investigating olfaction [7].

TAAR ligands are referred to as trace amines, a class of chemicals structurally and functionally similar to biogenic amines like the important neurotransmitters dopamine and serotonin [7, 8]. The expression patterns of TAARs within the olfactory epithelium and further organ systems, including the central nervous system, have attracted considerable attention due to their proposed role in mediating neurological processes [9]. Consequently, they are implicated in olfaction, and mediating olfactory signals to behaviour [7, 9, 10]. While the specific ligands for many TAARs remain unknown, confirmed ligands for certain TAARs include pheromones and other substances that convey social and behavioral cues [11–13]. These also include kairomones —substances that benefit the receiver of the signal rather than the sender in interspecies interactions. Examples of kairomones are predator-related signals perceived by mice, which help them detect and avoid potential threats [2, 6].

TAAR receptors are markedly less variable than classical ORs, but show distinct variability between species. While the genomic structure of TAARs is conserved across vertebrates, with nine distinct TAAR loci located on the same chromosome, various numbers of paralogues per locus can be present, and loci can be lost by pseudogenization [14]. An example of loss of TAAR genes is found in humans, where only six functional TAARs having been identified so far. Rodents, in contrast, gained additional TAARs, as mice and rats have been found to carry 15 and 17 TAARs, respectively. In zebrafish, 109 TAARs have been observed [6].

While TAAR diversity and function is best described in humans and model species [6], there is a growing body of knowledge on natural TAAR variability in various wildlife mammal species [15], including various primates [16], sac-winged bats (*Saccopterix bilineata*) [17] and racoons (*Procyon lotor*) [18], allowing for some extrapolation as to TAAR evolution. Generally, higher diversity is assumed to be linked to more intricate olfactory signalling within the species [19, 20]. This is similar to the assumptions concerning MHC diversity, where it is hypothesized that a more diverse MHC composition confers resistance against a broader range of pathogens, due to the limited number of antigens that can be presented by a single MHC allele’s binding site [21–23]. The question of which loci are polymorphic appears to be species-dependent [6, 10, 24]. However, given the limited information currently available on TAAR genetics, particularly in non-model species, the forces driving diversification and TAAR polymorphisms remain largely unknown.

Meerkats, as social mammals and cooperative breeders [25], are an intriguing species to study olfaction, since olfactory cues are presumed to play a major role in various aspects of meerkat behaviour, including foraging [26], social communication [27, 28] and territorial behaviours [29]. Meerkat display various scent marking behaviours which differ by sex and dominance status [28, 30]. Additionally, scent marks may carry a profound depth of individual information, like individual age, sex, group membership and dominance status [31–33], implying individual scent fingerprints. As small predators, meerkats are vulnerable to predation themselves, and previous studies have suggested the ability of meerkats to detect predator cues, i.e. kairomones, from excrements [34, 35]. Additionally, the neuroanatomy of the meerkat olfactory systems suggest adaptations to provide a very fine sense of smell and potentially specialization to detect TAAR-relevant ligands [36]. Nonetheless, to date, no detailed study on meerkat olfactory genetics has been conducted.

To address this gap, we leveraged the extensive dataset and large number of samples available from the Kalahari Meerkat Project [25] to establish a genotyping approach for characterizing polymorphic TAAR loci using high throughput sequencing. The objective of this study was to report the natural variability of meerkat TAARs, both at the population and individual level in this natural population that has been studied for over 20 years. This study will serve as the basis for subsequent investigations into the role of TAARs—and variation in TAAR diversity—in modulating the behaviour and mediating life history choices of a highly social mammal within an ecological setting.

## Results

### Polymorphism of the TAAR region in meerkats

**Fig. 1:**
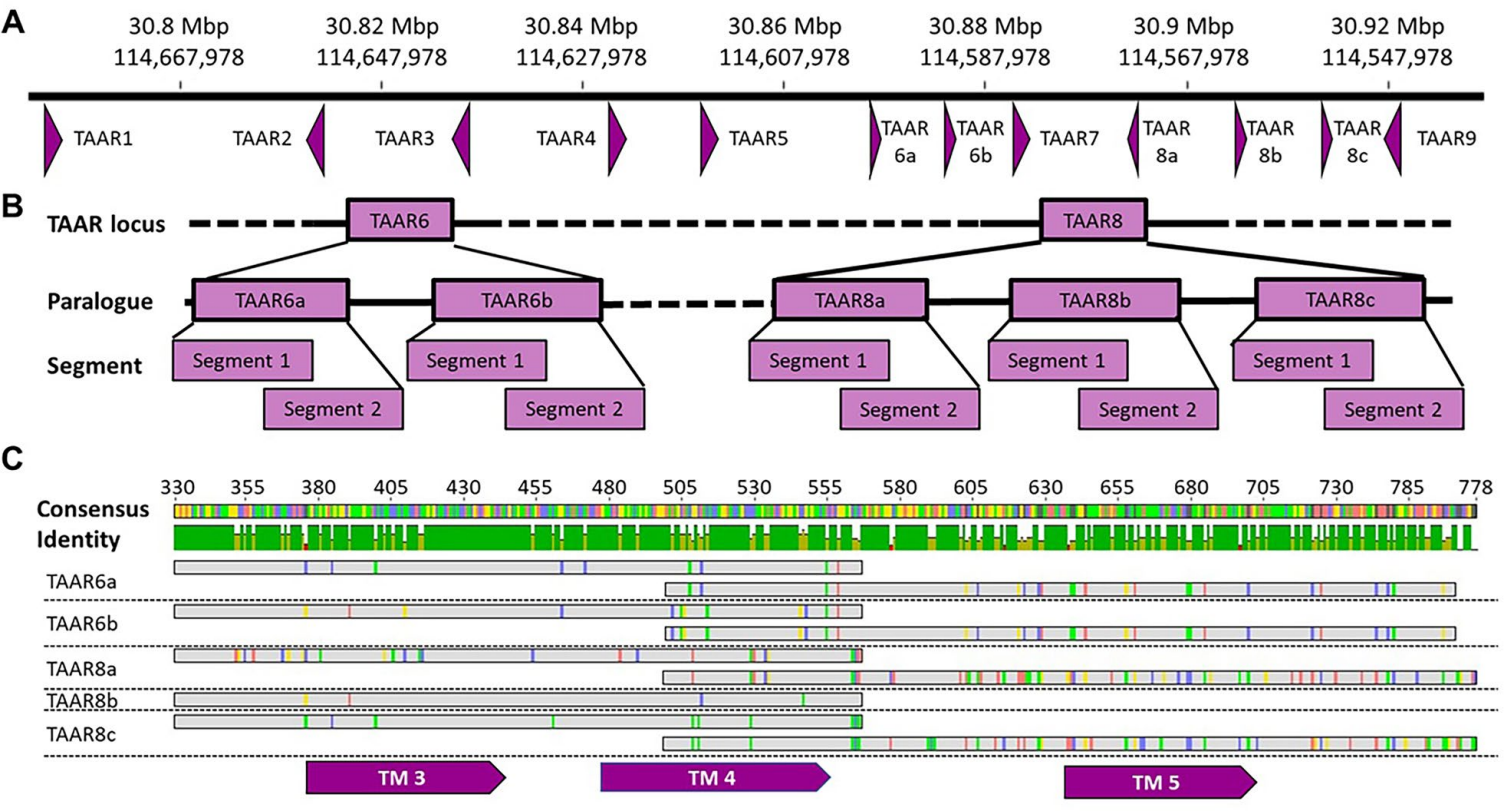
Schematic representation of the genetic structure and genotyping strategy for TAAR genes in meerkats. **A** Organization of the TAAR gene cluster on meerkat chromosome 7. Nine TAAR loci are located in ascending order within a ~ 135 kbp region of the ~ 145 Mbp chromosome. Gene orientation (purple arrows) and paralogues are indicated. Note that the chromosome is shown in reverse orientation. **B** Genotyping strategy illustrated for both TAAR loci targeted. Each TAAR paralogue (e.g., *TAAR6a/6b* and *TAAR8a/8b/8c*) was genotyped using two overlapping amplified fragments: segment 1 and segment 2. **C** Targeted TAAR6 and TAAR8 sequences. To capture the full range of variable sites, two overlapping segments were amplified for each paralogue. A single primer pair was used to amplify segment 1 across all five paralogues (*TAAR6a*, *6b*, *8a*, *8b*, *8c*). Due to sequence divergence, segment 2 required paralogue-specific primer pairs: one each for *TAAR6a/6b*, *TAAR8a/8c*, and *TAAR8b*. Positions are given according to the TAAR6a sequence in the reference genome. Primer pairs used are detailed in Table S5. Transmembrane domains are indicated by purple arrows. Sequences could be allocated to the respective paralogue based on the specific variable sites

**Fig. 2:**
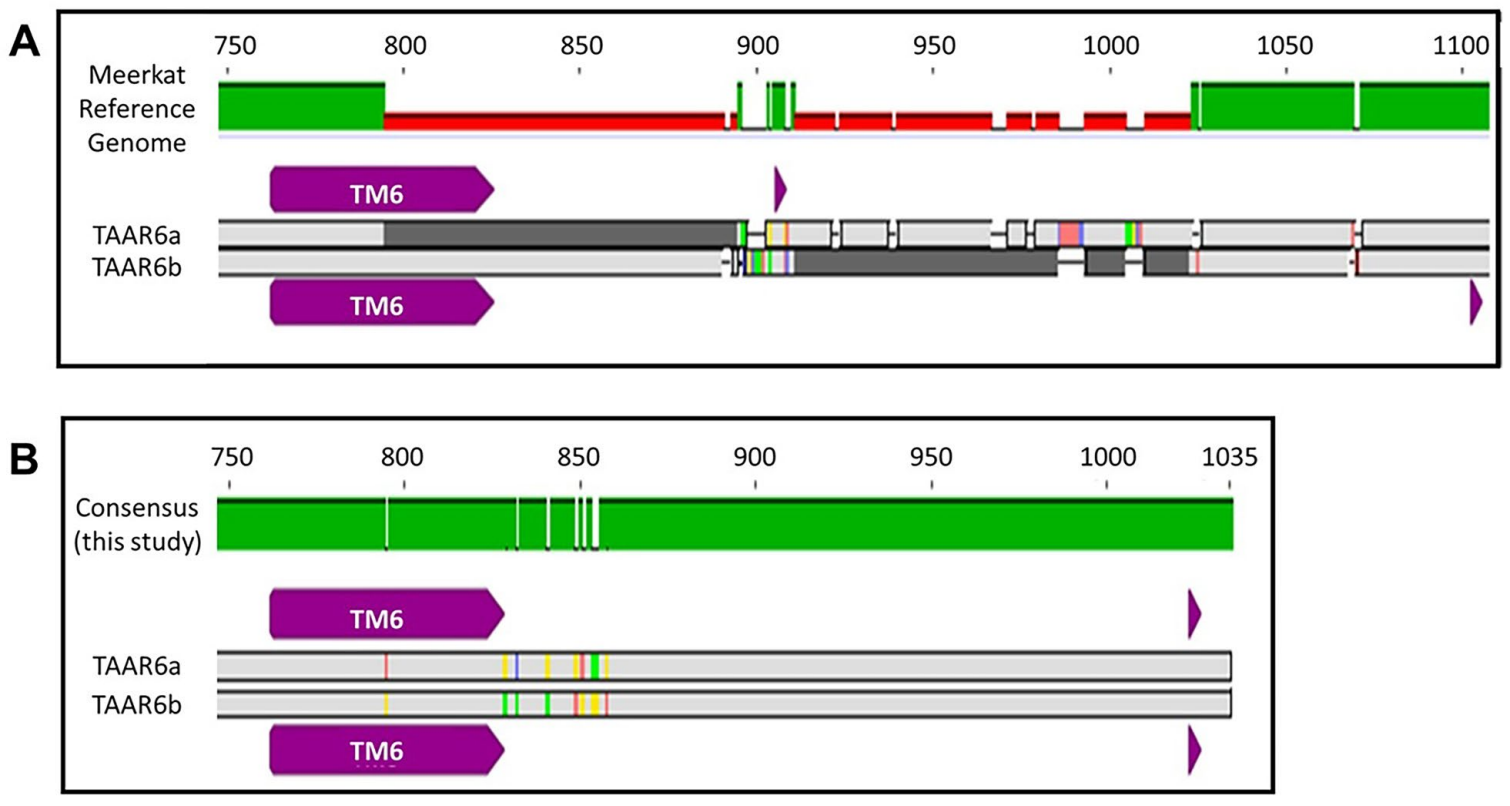
Comparison of partial TAAR6a and TAAR6b sequences from the meerkat reference genome and Sanger sequencing. **A** Partial TAAR6a and TAAR6b sequences from the meerkat reference genome (NCBI GenBank accession: PRJNA540846) contain unresolved base pairs (shown in dark grey) and sequence discrepancies between paralogues, which hindered the generation of a consensus sequence. **B** Sanger sequencing results from this study provided fully resolved sequences for both *TAAR6a* and *TAAR6b*, revealing that both genes are shorter than their reference genome counterparts. An open reading frame extends to position 1027, supporting the presence of functional TAAR6 genes. All previously ambiguous sites were resolved, enabling identification of variable positions and the construction of consensus sequences. Putative transmembrane domains (TM) are highlighted in purple. Start and stop codons are marked with purple arrows

A comprehensive survey of all TAARs (TAAR1-TAAR9) in 34 individual meerkats by Sanger sequencing revealed the presence of functional genes for all nine TAARs. It is noteworthy that only TAAR6 and TAAR8 exhibited genetic polymorphism in this species. The meerkat TAARs are located on chromosome 7, and all obtained sequences were mapped to the TAAR region from the meerkat genome available on GenBank® (accession number PRJNA540846), which was constructed using data from a single individual. The mapping of the sequences for TAAR6 and TAAR8 against this reference sequence revealed that meerkats carry two distinct paralogues for TAAR6, designated TAAR6a and TAAR6b, and three distinct paralogues for TAAR8, designated TAAR8a, TAAR8b and TAAR8c (Fig. 1A). Notably, the 1105 base pair GenBank® reference sequence for meerkat TAAR6 contains gaps and positions with missing nucleotide information. Furthermore, mismatches between the two TAAR6 paralogues between positions 780 and 1040 prevent the generation of a consensus sequences between both paralogues, and the reference sequence of TAAR6a contains a stop codon. The use of a specifically designed primer pair (519F-1152R, see materials and methods) targeting this sequence enabled the resolution of the ambiguities for both TAAR6 paralogues. Our findings demonstrate that, contrary to the published sequence, both TAAR6a and TAAR6b possess an open reading frame extending until the stop codon at position 1027. This resolves the aforementioned ambiguities as artefacts (Fig. 2). Consequently, both TAAR6 and TAAR8 were selected for subsequent high-throughput sequencing (Illumina) to assess population-wide and individual TAAR diversity in 398 meerkat individuals.

### Diversity of TAAR 6 and TAAR 8 in meerkats

To assess intra- and inter-individual TAAR6 and TAAR8 diversity in meerkats, we employed standard in-house Illumina MiSeq protocols [37, 38]. This resulted in the successful amplification of at least one TAAR paralogue for 398 individuals (see the materials and method section for details). To encompass all variable positions detected by Sanger sequencing, we conducted amplification of two overlapping segments, designated as segment 1 and segment 2, for each locus (Fig. 1B and C). Alleles were assigned to the specific TAAR6 or TAAR8 paralogues based on sequence similarity to the reference genome (see Fig. 1C).

**Fig. 3:**
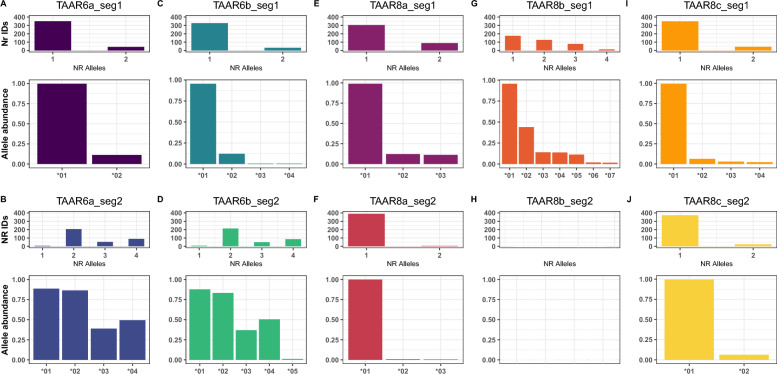
Allelic diversity and nucleotide allele frequencies of *TAAR6* and *TAAR8* paralogues in meerkats. For each *TAAR6* and *TAAR8* paralogue and segment (labeled **A**–**J**), the number of alleles per individual and the relative abundance of each nucleotide allele are shown. Allele abundance is expressed as the proportion of individuals carrying a given allele among those with successful amplification of the corresponding segment

For TAAR6a, out of a total of 398 individuals, 393 individuals were successfully genotyped at TAAR6a segment 1 and 354 individuals at TAAR6a segment 2. Two nucleotide alleles were identified for segment 1, with individuals carrying one to two (mean = 1.1 ± 0.3) distinct alleles (Fig. 3A). Four nucleotide alleles were identified for segment 2, with up to four (mean = 2.6 ± 0.9) distinct alleles per individual, indicating the presence of at least two gene copies at this TAAR6 paralogue (Fig. 3B). For segment 1, both nucleotide alleles code for distinct amino acid sequences (Table S1), with a substitution from valine (V) to lysine (L) at the amino acid position 139 (Table S1). Overall, 392 individuals (99.8%) carry TAAR_6a_1*01, corresponding to TAAR_6a_1_AA*01, whereas only 44 individuals (11.2%) carry TAAR_6a_1*02, corresponding to TAAR_6a_1_AA*02. For segment 2, two nucleotide alleles, TAAR_6a_2*02 and TAAR_6a_2*03, code for the same amino acid sequence TAAR_6a_2_AA*02, which is present in 351 individuals (99.2%). TAAR_6a_2*01, translating to TAAR_6a_2_AA*01, is present in 314 (88.7%), TAAR_6a_2*04, corresponding to TAAR_6a_2_AA*03, in 175 (49.4%) individuals (Fig. S1, Table S2). Here, variable positions are 225 with shift from glutamine (Q) to arginine (R), and 236 with a shift from serine (S) to glycine (G) (Table S1).

For TAAR6b, 360 and 354 individuals were successfully genotyped at segment 1 and segment 2, respectively. We detected four nucleotide alleles at segment 1, with individuals carrying one to two alleles (mean = 1.1 ± 0.3, Fig. 3C), and five nucleotide alleles for segment 2, with up to four nucleotide alleles (2.6 ± 0.9) per individual, indicating the presentence of at least to two gene copies of TAAR6b (Fig. 3D). The four nucleotide alleles at segment 1 code for two distinct amino acid alleles, with TAAR_6b_1*01, TAAR_6b_1*03 and TAAR_6b_1*04 translating into the same amino-acid sequence TAAR_6b_1_AA_*01, which is present in 344 individuals (95.6%). The remaining allele TAAR6b_1*02, translating into TAAR6b_1*AA*02, is present in 44 individuals (12.2%) (Fig. S1, Table S2). Between the amino acid sequences, there is a substitution from phenylalanine (F) to valine (V) at amino acid position 121 and from valine (V) to leucine (L) at position 139 (Table S1).

The five nucleotide alleles at segment 2 translate into four distinct amino acid alleles, with TAAR6b_2*02 and TAAR6b_2*03 translating into TAAR6b_2_AA*02, present in 341 individuals (96.3%), TAAR6b_2*01, translating into TAAR6b_2_AA*01, present in 311 individuals (87.9%), TAAR6b_2*04, translating into TAAR6b_2_AA*03, present in 179 individuals (50.6%) and TAAR6b_2*05, translating to TAAR6b_2_AA*04, detected in only 4 individuals (1.1%) (Fig. S1, Table S2). Variable positions in amino acids revealed a shift from glycine (G) to valine (V) at position 189, glutamine (Q) to arginine (R) at position 225 as well as substitutions from serine (S) to glycine (G) at position 236 (Table S1).

For TAAR8a, 393 and 394 individuals were successfully genotyped for segment 1 and 2, respectively, with three distinct nucleotide alleles detected for both segments. Individuals carried one to two alleles for both segments (segment 1: mean = 1.2 ± 0.4, segment 2: mean = 1.0 ± 0.1), indicating at least one gene copy for TAAR8a (Fig. 3E and F). Similar to the patterns of TAAR6, two nucleotide alleles of segment 1, TAAR8a_1*01 and TAAR8a_1*02, translated into the same amino acid sequence, TAAR8a_1_AA*01, which was present in 392 individuals (99.7%), whereas the third allele TAAR8a_1*03, translating into TAAR8a_1_AA*02, was markedly rarer (44 individuals, 11.2%) (Fig. S1 and Table S2). Between the amino acid alleles, we find a substitution from glycine (G) to serine (S) at amino acid position 154 (Table S1).

For segment 2, all nucleotide alleles translated into distinct amino acid alleles, with allele TAAR8a_2*01 (TAAR8a_2_AA*01) present in all 394 individuals (100%) and the two additional alleles, TAAR8a_2*02 (TAAR8a_2_AA*02) and TAAR8a_2*03 (TAAR8a_2_AA*03) markedly rarer, being present in 3 (0.8%) and 2 (0.5%) individuals, respectively (Fig. S1, Table S2). Variable positions in amino acids revealed a shift from cysteine (C) to phenylalanine (F) at position 186 as well as a substitution from aspartate (D) to glutamate (E) at position 197 (Table S1).

For TAAR8b, segment 1 was successfully TAAR-typed in 393 individuals, but no alleles detected in our analyses could be assigned not TAAR8b segment 2. The absence of distinct sequences for TAAR8b segment 2 is likely due to a methodological issue, where the primer pair used for this segment failed to amplify the targeted sequence in the multiplex reaction with primers for TAAR8a and TAAR8c, for unknown reasons. Of all the segments analysed, TAAR8b segment 1 showed the highest nucleotide diversity, with seven distinct alleles identified. Individuals carried up to four alleles (mean = 1.8 ± 0.9), suggesting two distinct TAAR8b gene copies in meerkats (Fig. 3G). The seven nucleotide alleles translate into five distinct amino acid alleles, with TAAR8b_1_AA*01 (i.e., TAAR8b_1*01 and TAAR8b_1*03) being the most prevalent, present in 380 individuals (96.7%). Nucleotide alleles TAAR8b_1*05 and TAAR8b_1*07 coded for the same amino acid allele TAAR8b_1_AA*04, occurring in 48 individuals (12.2%). Of the remaining three alleles, TAAR8b_1*02 (TAAR8b_1_AA*02) was the most frequent (173 individuals, 44%), TAAR8b_1*04 (TAAR8b_1_AA*03) showed intermediate prevalence (54 individuals, 13.7%), and TAAR8b_1*06 (TAAR8b_1_AA*05) was rare, being present in only seven individuals (1.8%) (Fig. S1 and Table S2). Variable positions across the amino acid alleles were characterized by substitutions from valine (V) to threonine (T) at amino acid position 113, alanine (A) to valine (V) at position 114, phenylalanine (F) to tyrosine (Y) at position 125, valine (V) to leucine (L) at position 139, threonine (T) to methionine (M) at amino acid position 145, glycine (G) to glutamate (E) at position 150, isoleucine (I) to phenylalanine (F) at 153, tryptophan (W) to arginine (R) at position 157 and finally alanine (A) to valine (V) at position 172 (Table S1).

For TAAR8c, 393 and 395 individuals were successfully sequenced at segment 1 and segment 2, respectively. For segment 1, four nucleotide alleles were detected, with individuals carrying up to two (mean = 1.1 ± 0.3) (Fig. 3I). Of these four alleles, three—TAAR8c_1*01, TAAR8c_1*03 and TAAR8c_1*04—translate into the same amino acid sequence, TAAR8c_1_AA*01, which is almost ubiquitous, being present in 391 individuals (99.5%). The remaining nucleotide allele, TAAR8c_1*02, translating into TAAR8c_1_AA*02, is again rare and detected in only 25 individuals (6.4%) (Fig. S1 and Table S2). Both amino acids differ in one amino acid, glycine (G) to arginine (R) at amino acid position 150 (Table S1).

Segment 2 contains two nucleotide alleles, with individuals carrying one or both (mean = 1.1 ± 0.2, Fig. 3J). Both code for two distinct amino acid alleles, with TAAR8c_2*01 (TAAR8c_2_AA*01) present in 393 individuals (99.5%) and TAAR8c_2*02 (TAAR8c_2_AA*02) present in only 25 individuals (6.3%) (see Fig. S1, Table S2). Both vary in position 182, carrying either serine (S) or cysteine (C) at this position (Table S1).

### Temporal variation of TAAR6 and TAAR8 across more than 20 years

**Fig. 4:**
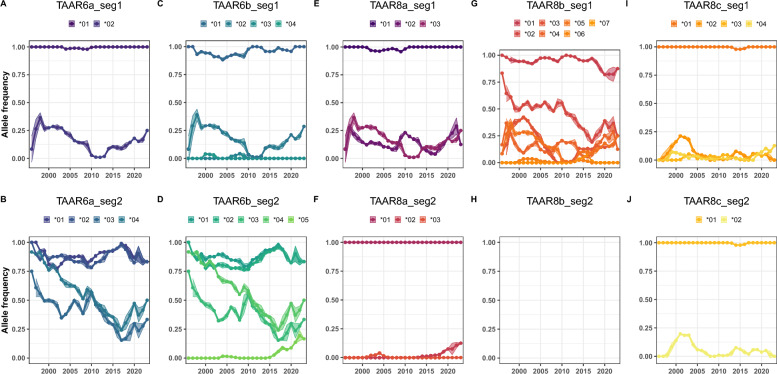
Temporal variation in nucleotide allele abundance of *TAAR6* and *TAAR8* paralogues over 20 years. The relative abundance of each segment of the *TAAR6* and *TAAR8* paralogues is shown as the mean ± standard deviation for four three-month periods (Jan-Mar, Apr-Jun, Jul-Sep, Oct-Dec) each year. Data points represent the mean abundance across individuals alive during each respective period

**Fig. 5:**
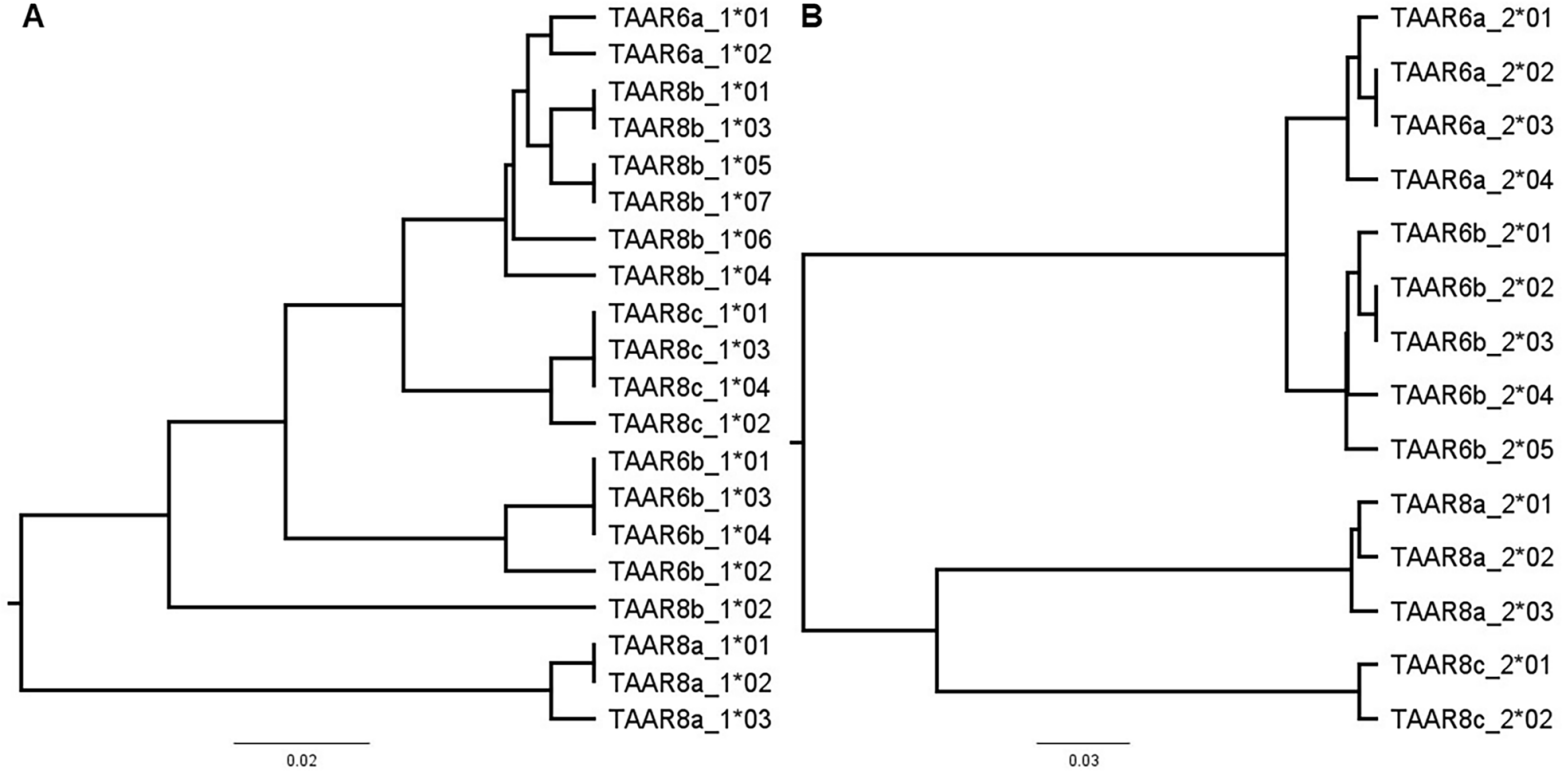
Phylogenetic analysis of *TAAR6* and *TAAR8* amino acid sequences. Phylogenetic trees were constructed in Geneious (Version 11.0.4) using the Jukes-Cantor genetic distance model and the UPGMA method with 1000 bootstrap replications. Separate trees are shown for **A** segment 1 and **B** segment 2 of the *TAAR6* and *TAAR8* alleles

The general pattern observed for both TAAR loci and across all genotyped paralogues was the presence of one nucleotide allele being very common (Fig. 4), mirrored by the presence of one amino acid allele in almost all individuals (Figs. S1, S2). This could be due to an almost ubiquitous nucleotide allele or multiple nucleotide alleles encoding the same amino acid (Fig. 5), with up to three nucleotide alleles coding for the same amino acid across both segments of TAAR6 and TAAR8. This relatively high level of synonymous substitutions, combined with the presence of 14 sites under purifying selection in segment 1 and 17 sites in segment 2 (Table S1), identified through Fixed Effects Likelihood (FEL) analyses [39], suggests strong selective pressure on TAAR6 and TAAR8 to preserve specific amino acids at specific positions. In addition to the highly conserved alleles, rarer alleles were found in some individuals. This pattern remained constsistent across the more than 20 years considered in the study. The additional, variable nucleotide and amino acid alleles demonstrate marked fluctuation across the study period (Fig. 4, Fig. S2). Notably, four nucleotide alleles—TAAR6a_1*02, TAAR6b_1*02, TAAR8a_1*03 and TAAR8b_1*05—are jointly present in the same 44 individuals, resulting in identical trajectories and providing compelling evidence that these alleles form a single haplotype (Fig. 4). Similarly, nucleotide allele pairs TAAR6a and TAAR6b segment 2 *01 through *04 are almost always present together (Fig. S3), resulting in very similar, though not perfectly matched, trajectories (Fig. 4).

## Discussion

This study represents the first investigation into the natural diversity and variability of TAAR receptors in wild meerkats. Two loci, TAAR6 and TAAR8, were identified as polymorphic in meerkats. Our results align with the presence of two paralogues of TAAR6 and three paralogues of TAAR8 in the meerkat reference genome used in this study. Additionally, multiple different alleles were identified for all paralogues, with individuals carrying up to four alleles at one or more paralogues. These findings suggest the presence of copy number variations at TAAR6 and TAAR8 and indicate that individuals exhibit greater diversity than previously known. However, as the reference genome is based on a single individual, further polymorphisms at other TAAR loci cannot be excluded, and we may not have captured the full diversity of TAARs in meerkats.

Both polymorphic loci identified by our analyses are characterised by the simultaneous presence of multiple paralogues, specifically TAAR6a and TAAR6b, TAAR8a, TAAR8b and TAAR8c. There is considerable inter-individual variability, with individuals carrying up to four different alleles at TAAR6a, TAAR6b and TAAR8b, which suggests the presence of at least two different gene copies for these paralogues. The sequencing of two segments for each locus revealed the presence of at least four distinct nucleotide alleles at two gene copies for TAAR6a, at least five distinct nucleotide alleles at two gene copies for TAAR6b, at least three distinct nucleotide alleles at one gene copy for TAAR8a, at least seven distinct nucleotide alleles at two gene copies for TAAR8b, and at least four distinct nucleotide alleles at one gene copy for TAAR8c. In general, a notable combination of variability and conserved sites is observed in both TAAR6 and TAAR8. Across all five paralogues, an almost ubiquitous nucleotide and amino acid allele is consistently present, carried by more than 95% of successfully genotyped individuals, combined with other alleles that vary markedly in abundance. At both the nucleotide and amino acid levels, the more common additional alleles are present in almost 90% of the TAAR-typed meerkats, while the rarest are found in less than 1% of TAAR-typed meerkats.

Despite using overlapping segments, our approach did not allow us to determine the exact number of alleles per paralogue. This limitation arises because variable sites within each paralogue are not present in the overlap between sequences of both segments. As a result, two or more variable sequences for one segment could share the same sequence for the other, which may explain the higher similarity of trajectories across segments of different paralogues rather than between segments of the same paralogue. To determine the exact number of alleles per paralogue, a different approach using longer reads would be required. Additionally, amplification biases inherent in PCR methods may lead to an underestimation of TAAR diversity if specific alleles fail to amplify, as observed for TAAR8b segment 2. Nonetheless, our study provides strong evidence of significant meerkat TAAR diversity and is more likely to underestimate, rather than overestimate, individual variability.

For each TAAR locus, we identified synonymous substitutions in at least one segment, with up to three different nucleotide alleles translating into the same amino acid allele. This pattern leads to the almost ubiquitous presence of specific amino acid alleles across the five assessed paralogues, which strongly suggests a high level of selective pressure on these amino acid sequences. This high level of purifying selection is further supported by the presence of multiple sites under purifying selection across both segments of the analyzed TAAR6 and TAAR8 paralogues. It can be surmised that the conservation of these sequences and specific stereochemical properties at certain positions is crucial for maintaining the functionality of the respective paralogue and potentially for ligand binding. Currently, the expression patterns and, consequently, the precise function of most TAARs in non-model species remain unknown. Although TAAR expression patterns across various tissues have yet to be explored in meerkats, the expression of certain TAAR receptors in the central nervous system of other mammals [9] suggests that these highly conserved alleles may play a role beyond olfaction, potentially mediating additional central functions related to trace-amine signaling [7, 12].

In addition to these conserved patterns, we find marked variability at the TAAR level between individuals, including copy number variation. It is hypothesised that gene duplication may result in neofunctionalization of both TAAR6 and TAAR8, which could potentially enhance the olfactory abilities of carriers [40]. Receptor diversity may facilitate the intricate process of odour recognition, as specific odours often engage multiple receptors [20]. In light of the findings in rodents, where the deletion of TAAR4 directly resulted in a loss of avoidance of certain predator cues [19], we propose that the presence of additional TAAR6 and/ or TAAR8 alleles could lead to differences in behaviour and olfactory ability between individuals.

Detailed insights into total and inter-individual TAAR diversity in non-model species are largely lacking. Nevertheless, considerable efforts over the past two decades have advanced our understanding of TAAR evolution, leading to a general comprehension of TAAR phylogeny and interspecies variation [15, 16, 24, 41, 42] A comparison of the TAAR diversity reported here for meerkats, namely 12 different TAAR paralogues with up to 16 functional TAAR genes, with that of further mammalian species reveals a close resemblance to rodents, as mice and rats are reported to carry 15 and 17 functional TAARs, respectively [6, 10]. While both ungulates and marsupials have been reported to carry slightly more functional TAARs (reviewed in [10, 24]), primates in particular are characterised by a loss of functional TAARs through pseudogenisation [14, 16], resulting in the retention of only six functional TAARs in humans, with a further reduction in other apes. The documented importance of TAARs for socio-sexual communication and predator detection in rodents [11, 12, 43], and the reported olfactory abilities of meerkats, including social signalling and predator detection [29, 31, 35], suggest that TAARs may play a similar role in mediating meerkat odour-guided behaviours as they do in rodents.

The reported TAAR structure provides further evidence for the involvement of TAAR6 and TAAR8 in meerkat olfaction. We find that meerkat TAAR6 and TAAR8 both contain the so-called amine recognition motifs in transmembrane region 3 (TM3), which has been reported as a conserved odorant contact site. In particular, the aspartic acid (D) at position 112, which is also conserved in meerkat TAAR6 and TAAR8, has been described as forming a salt bridge with the amino group located in ligands [13, 44]. Amino acid substitutions that alter the stereochemical characteristics, specifically the replacement of serine or cysteine with tyrosine at position 132, have been observed to affect ligand binding, resulting in a shift from N,N-dimethylphenylethylamine to N,N-dimethylcyclohexamine [45]. Similarly, alleles differing in their stereochemistry have been identified across the polymorphic meerkat TAARS. For instance, at segment 1 of TAAR6a, where a substitution from glutamine (Q) to arginine (R) at amino acid position 225 results in a change from a neutral to a positive net charge. The substitution of glycine (G) to serine (S) at position 236 results in a shift from non-polar to polar bonds. Similarly, segment 1 of TAAR8a contains amino acids with distinct stereochemistry at position 186, namely either the Brønsted acid cysteine (C) or the non-polar phenylalanine (F). TAAR8c contains two distinct amino acid sequences in segment 1 with a change from the non-polar and neutral glycine (G) to the basic polar and positively charged arginine (R) at position 150. The amino acid sequences of segment 2 exhibited considerable variation in the substituted position 182, ranging from serine (S), a polar amino acid, to cysteine (C), a Brønsted acid. The potential impact of these changes in stereochemistry on meerkat TAAR ligand affinity and their behavioural implications remain to be elucidated. However, it is highly probable that variability in ligand binding in meerkats carrying additional TAAR6 or TAAR8 alleles will be observed.

The distribution of rarer TAAR alleles within the meerkat population exhibits notable changes over the course of the present study, extending over a period of more than 20 years. Some of the variable alleles demonstrate pronounced fluctuations across multiple years. In light of the highly skewed reproductive system of meerkats, with a small number of individuals largely monopolising reproduction [25, 46, 47], marked differences across time are to be expected. The question of whether TAAR diversity conveys fitness benefits and the underlying mechanisms responsible for these potential benefits remains to be answered. Higher TAAR diversity has been linked to mate choice in bats [17] and raccoons [18], facilitating the selection of mates with greater MHC diversity. Given that MHC composition is associated with pathogen susceptibility and resilience in the same population [48], exploring whether TAAR diversity influences life history decisions, such as group or mate choice, and whether these choices enhance meerkat fitness by improving offspring’s ability to handle infectious diseases, represents an intriguing avenue for future research.

In conclusion, the methods presented here for more detailed investigations of TAARs have significant potential to generate valuable insights into the mechanisms behind odour-guided behaviours, including in non-model species. It is our hope that researchers will be encouraged to incorporate TAAR genomics into studies of socio-sexual communication, predator avoidance and life history of known macrosmatic wildlife species.

## Methods

### Sample collection

Since 1993, tail tip samples from wild, fully habituated meerkats have been routinely collected as part of the long-term Kalahari Meerkat Project in the Kuruman River Reserve in Northern South Africa (see [25] for details), usually upon first emergence of pups from their natal burrow (for details see [46, 49]). DNA was extracted from the tail tips at the Institute of Evolutionary Biology, School of Biological Sciences (University of Edinburgh, UK) and the Department of Evolutionary Biology and Environmental Studies (University of Zurich, Switzerland), and aliquots of samples were transported to the Institute of Evolutionary Ecology and Conservation Genomics at Ulm University (Germany) on ice for TAAR genotyping, where they are kept at -20 °C until analysis.

The individuals selected for the study were alive between 1996 and 2023 and documented as members of groups that persisted for a minimum of two years [50]. To assess TAAR diversity, we included (i) males that were recorded in multiple groups, and (ii) reproductive females, defined as females with records of having given birth regardless of pup survival, resulting in n = 398 individuals. We also extracted individual metadata, namely first (birth or immigration into the study population) and last (death or disappearance) date from the Meerkat Project Database (for details see [50]). Population size and number of successfully TAAR-genotyped individuals are presented in Table S3.

### Assessment of TAAR polymorphism in meerkats using Sanger sequencing

Since TAAR loci vary in polymorphism, we used Sanger sequencing of seven to ten individuals to identify the presence and location of polymorphisms in TAAR loci 1 to 9 in the meerkat population. Briefly, for each primer pair (see Table S4), we prepared 10 µl PCR reactions, containing 5 µl AmpliTaq^TM^Gold 360 Master Mix (Thermo Fisher Scientific), 0.3 µl of forward and reverse primer, 1 µl of sequencing buffer and 2.4 µl of purified water and 1 µl sample DNA. Cycling conditions were set to an initial denaturation at 95 °C for 10 min, followed by 35 cycles of denaturation at 95 °C for 30 s, annealing at the respective annealing temperature for the primer pair (see Table S4) for 30 s, and elongation at 72 °C for 45 s, followed by a final elongation at 72 °C for 10 min. PCR success was confirmed by gel electrophoreses on a 1.5% agarose gel.

PCR products were prepared for Sanger sequencing by performing a two-component clean-up by the FastAP/Exo treatment according to manufacturer’s instructions (Thermo Fisher Scientific™). Cleaned PCR products were sequenced using the BigDye™ Terminator v3.1 Cycle Sequencing Kit (Applied Biosystems™) following manufacturer’s instructions, with one reaction run for both forward and reverse direction per sample, containing 2 μl of the purified PCR product, 1 μl BigDye™ reaction mix, 2 μl BigDye™ sequencing buffer, 4 μl purified water, and 1 μl of either forward or reverse primer. Sequencing PCRs were performed with 30 cycles of denaturation at 95 °C for 10 s, annealing at 52 °C for 15 s and elongation at 60 °C for 2 min, followed by a final elongation at 72 °C for 1 min PCR. The sequencing PCR products were then purified using a BigDye XTerminator™ Purification Kit (Applied Biosystems™) according to manufacturer’s instructions and sequenced on an ABI 3130 Genetic Analyzer (Applied Biosystems™). The resulting raw sequences were analysed in Geneious (versions 11.0.4 and 11.1.5), with TAAR1 to TAAR5, TAAR7 and TAAR9 being monomorphic according to our results, although the existence of further polymorphic TAARs cannot be excluded in meerkats. In contrast, polymorphisms were detected for TAAR6 and TAAR8, which were subsequently followed up by establishing high-throughput sequencing protocols using the Illumina platform.

### Assessment of TAAR 6 and TAAR 8 diversity in meerkats by Illumina high throughput sequencing

In order to target the functional units of the polymorphic TAAR6 and TAAR8 loci, primers were designed using the consensus of TAAR6 and TAAR8 sequences of *S. suricatta* encoded on chromosome 7, as available in GenBank® (genome dataset VVHF042, https://www.ncbi.nlm.nih.gov/nuccore/ 1700039957), using Primer3 in Geneious (version 11.0.4, http://www.geneious.com) in conjunction with the sequences generated by our Sanger sequencing. We tested a number of primer pairs for both joint and separate amplification of TAAR6 and TAAR8 (see Table S5) and optimal amplification conditions (number of cycles, annealing temperatures) in a subset of 34 (out of 398) meerkat samples. The comprehensive primer testing resulted in a joint primer pair for segment 1 of both loci and three distinct primer pairs targeting segment 2 of TAAR6, TAAAR8a/c and TAAR8b, specifically TAAR6-8F and TAAR6-8R for segment 1 of all TAAR6 and TAAR 8 paralogues, 432F and T6-741R for segment 2 of TAAR6, 432F and T8b-741 for segment 2 of TAAR8b, 478F and 802R for segment 2 of TAAR8a and TAAR8c (Table S5). Primers were designed to include the transmembrane domains three through five of the respective TAARs and cover the variable positions within and between paralogues. This allowed us to sort resulting sequences to the respective paralogue. Based on specific variable positions present in each paralogue (see Fig. 1C), we could validate the specificity of the primer pairs by comparing the resulting sequences to the reference genome and the sanger sequences.

For high throughput sequencing of all study animals included in the present study (n = 398), we prepared three PCR reactions per sample, one with the primer pair TAAR6-8F/ TAAR6-8R, one with the primer pair 478F/802R and a multiplex reaction including the forward primer 432F and both reverse primers T6-741R and T68b-741R, including 32 randomly chosen technical replicates for each primer pair. PCR reactions of overall 10 µl volume contained 5 µl AmpliTaq^TM^Gold 360 Master Mix (Thermo Fisher Scientific), 0.3 µl respectively of the locus-specific forward and reverse primer, 1 µl of sequencing buffer, 2.4 µl of purified water and 1 µl of sample DNA. Cycling conditions were initial denaturation at 95 °C for 10 min, 30 cycles of denaturation at 95 °C for 30 s, annealing at 58 °C for 30 s and elongation at 72 °C for 45 s, followed by a final elongation step at 72 °C for 7 min at the end of the PCR cycle. Successful amplification was assessed using gel electrophoreses as described above.

Following PCR, we used standard barcoding protocols. Briefly, we prepared 20 µl volume barcoding PCR reactions, containing 10 µl of AmpliTaq^TM^Gold 360 Master Mix, 1 µl of sequencing buffer, 3 µl of purified water, 4 µl of the specific barcode (Standard Bio Tools®) and 2 µl of template DNA. Cycling conditions for barcoding PCRs were initial denaturation at 95 °C for 10 min, followed by 8 cycles of denaturation at 95 °C for 30 s, annealing at 60 °C for 30 s and elongation at 72 °C for 45 s, followed by a final elongation step at 72 °C for 7 min. Barcoded samples were purified by the NucleoMag® NGS Clean-up and Size Select kit (Macherey–Nagel®) following the manufacturer’s instructions. We used a DNA intercalating fluorescent dye method (QuantiFluor® dsDNA System, Promega) and absorbance measurement (F200 plate reader, Tecan) for DNA quantification according to manufacturer’s instructions, and normalized our samples to a final library concentration of 6 nM. Sequencing was performed on an in-house Illumina Miseq System with a Reagent Kit v2, 500 cycles, using standard settings.

### Bioinformatic quality filtering, TAAR allele calling and individual genotyping

Following Illumina sequencing, we used the ACACIA pipeline [51] for quality filtering and allele calling using default settings. Within processing the pipeline, paired-end reads were merged, sequences not containing complete primer sequences excluded and primer sequences were cut from the sequences. Quality filtering was applied, and only sequences with a Phred score of 30 or more, indicating a base call accuracy of at least 99.9%, in at least 90% of the nucleotide positions were included in further analyses. Chimeric sequences were removed and sequences compared to an internal database consisting of 33 Feliformia TAAR6 and TAAR8 sequences downloaded from NCBI GenBank® on 29th April 2022 (Search Terms: Trace[All Fields] AND amine[All Fields] AND associated[All Fields] AND 6/8[All Fields] AND (“Feliformia”[Organism] OR feliformia[All Fields]) using BLAST. Final TAAR allele calling was performed using the Oligotyping algorithm implemented in ACACIA.

Following the bioinformatic pipeline, we compared technical replicates of the same individual, and retained only the sample with the highest coverage among the replicates. We further excluded alleles that failed to be called in both replicates, and all alleles with less than 200 read coverage or 2% of reads in the respective sample.

Illumina sequencing generated 18,732,276 raw reads in a single run across all primer pairs, which were subsequently sorted and processed by primer pair. For each primer pair, coverage and repeatability between technical replicates are detailed in the supplement (Table S6). Nucleotide alleles were allocated to their respective paralogue in each locus in Geneious 11.0.4 (http://www.geneious.com), translated and aligned using MAFFT [52]. Phylogenetic trees were generated by the inbuilt tree builder in Geneious, using Jukes-Cantor as genetic distance model, UPGMA as tree build method and 1000 bootstrap replications. To test for evidence of diversifying or purifying selection, we conducted two Fixed Effects Likelihood (FEL) analyses—one for all nucleotide alleles in segment 1 and segment 2 of both TAAR loci—using the Datamonkey bioinformatics web application [53]. FEL, which uses a maximum-likelihood (ML) approach to infer nonsynonymous (dN) and synonymous (dS) substitution rates on a per-site basis while accounting for phylogeny, is the recommended test for detecting pervasive selection at the site level in small datasets [39, 53]. Datasets were curated and descriptive statistics extracted using the functions of the library “tidyverse” [54] in R (version 4.2.2 and 4.3.2) ( [55]) in R Studio [56]. Graphics were created using the package ggplot2 [57] and Geneious.

## Supplementary Information

Additional file1

Additional file2

Additional file3

Additional file4

Additional file5

## Data Availability

Data used in this study is available at Github: https://github.com/Nadine-MK/Meerkat-TAAR-characterization.

## Declarations

### Statement on inclusion

The fieldwork for this study was conducted at the Kalahari Meerkat Project (KMP) in the remote Kalahari Desert of South Africa. All fieldwork activities were discussed with and approved by the local stakeholders of the Kuruman River Reserve. As is customary at the KMP, our research was carried out by an internationally diverse team, including individuals from South Africa, to whom we are deeply grateful. The laboratory and analytical phases of the study were conducted in Germany, and this publication reflects the collaborative efforts of authors from multiple countries.
