# Supplementary figures and images for: Natural variability of trace-amine associated receptors in wild meerkats
Source: Front Zool. 2025 Nov 27;22:37. doi: 10.1186/s12983-025-00590-2 (PMC12659089; doi:10.1186/s12983-025-00590-2)

Presence 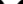 0 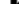 1

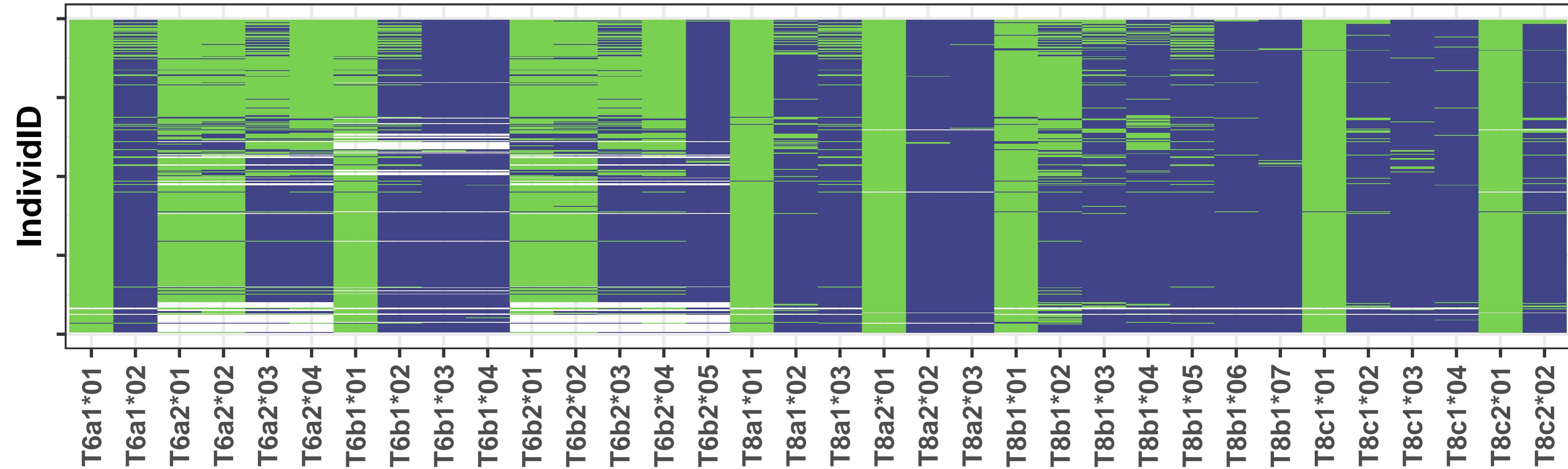

Supplement: Supplementary file 1 — Additional file1 [file 12983_2025_590_MOESM1_ESM.pdf]

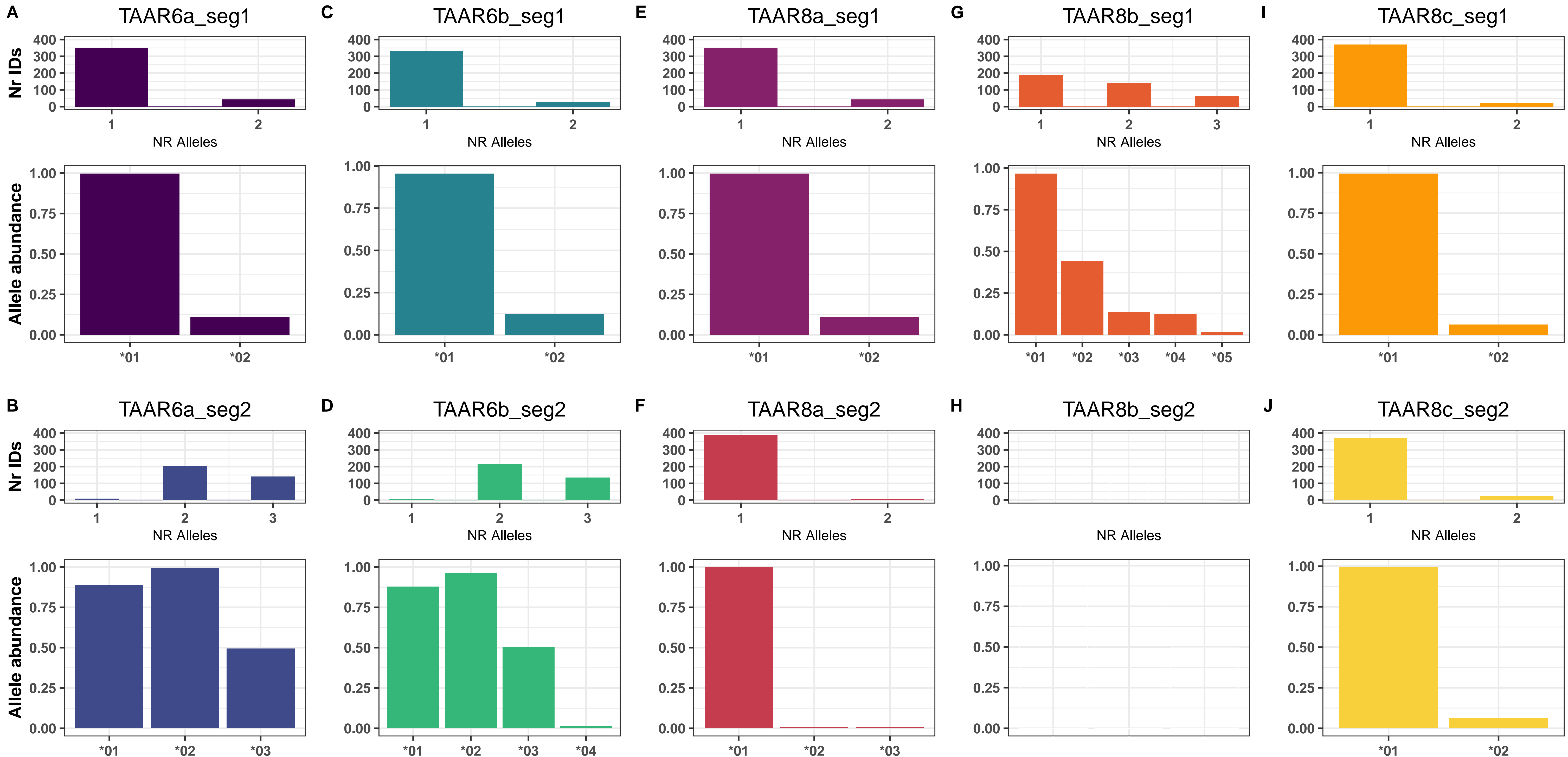

Supplement: Supplementary file 4 — Additional file4 [file 12983_2025_590_MOESM4_ESM.pdf]

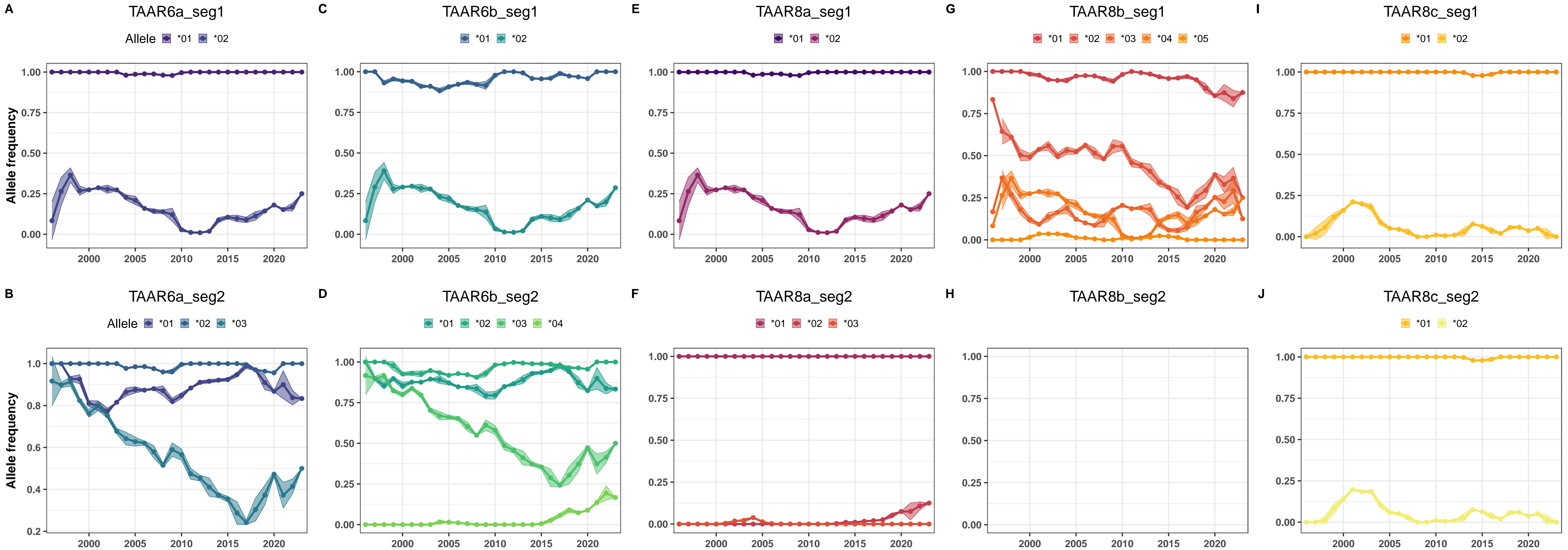

Supplement: Supplementary file 5 — Additional file5 [file 12983_2025_590_MOESM5_ESM.pdf]
