# Supplementary material for: Natural variability of trace-amine associated receptors in wild meerkats
Source: Front Zool. 2025 Nov 27;22:37. doi: 10.1186/s12983-025-00590-2 (PMC12659089; doi:10.1186/s12983-025-00590-2)
Supplement: Supplementary file 2 — Additional file2 [file 12983_2025_590_MOESM2_ESM.docx]

**Supplement**

**Supplementary Figure legends**

**Figure S1:** Amino acid allele abundances of TAAR6 and TAAR8 paralogues. For each paralogue and segment, the abundance was calculated as the proportion of individuals carrying a specific allele, relative to the total number of individuals with successful amplification of the respective segment.

**Figure S2:** Amino acid allele relative abundance of TAAR6 and TAAR8 paralogues across > 20 years. Relative abundances of each segment of TAAR6 and TAAR8 paralogues are depicted as mean ± sd relative abundance across four three-months periods (Jan-Mar, Apr-Jun, Jul-Sep, Oct-Dec) for each year, counting individuals alive in the respective three-months period.

**Figure S3:** Visualization of TAAR6 and TAAR8 nucleotide alleles present in TAAR-typed individuals.

**Supplementary Tables**

**Table S1:** Amino acid sequences of sequenced TAAR6 and TAAR8 paralogues across both segments. For each segment, the positions are given as position within the respective segment and according to the reference genome. Sites under purifying selection are indicated (X) for conserved sites (in blue) and at variable sites (in yellow). Sites that are both variable and under purifying selection are coloured in green. Table provided as separate Excel-file.

| **Table S2:** Nucleotide and amino acid allele diversity and abundance at TAAR6 and TAAR8 across segments 1 and 2. Up to three nucleotide [NT] alleles code for the same amino acid [AA] allele. Consequently, relative abundances of both nucleotide [N] and amino acid alleles are given separately. | | | | | | |
| --- | --- | --- | --- | --- | --- | --- |
| **Primer Pair** | **Segment** | **NT Allele** | **N at Segment** | **NT Allele Abundance** | **AA Allele** | **AA Allele Abundance** |
| **TAAR6a** | | | | | | |
| TAAR6-8F & TAAR6-8R | Seg1 | TAAR6a_1*01 | 393 | 99.7% | TAAR6a_1_AA*01 | 99.7% |
| TAAR6-8F & TAAR6-8R | Seg1 | TAAR6a_1*02 | 393 | 11.2% | TAAR6a_1_AA*02 | 11.2% |
| 432F &T6-741R | Seg2 | TAAR6a_2*01 | 354 | 88.7% | TAAR6a_2_AA*01 | 88.7% |
| 432F &T6-741R | Seg2 | TAAR6a_2*02 | 354 | 86.4% | TAAR6a_2_AA*02 | 99.2% |
| 432F &T6-741R | Seg2 | TAAR6a_2*03 | 354 | 39% |  |  |
| 432F &T6-741R | Seg2 | TAAR6a_2*04 | 354 | 49.4% | TAAR6a_2_AA*03 | 49.4% |
| **TAAR6b** | | | | | | |
| TAAR6-8F & TAAR6-8R | Seg1 | TAAR6b_1*01 | 360 | 95.6% | TAAR6b_1_AA*01 | 95.6% |
| TAAR6-8F & TAAR6-8R | Seg1 | TAAR6b_1*03 | 360 | 0.6% |  |  |
| TAAR6-8F & TAAR6-8R | Seg1 | TAAR6b_1*04 | 360 | 0.6% |  |  |
| TAAR6-8F & TAAR6-8R | Seg1 | TAAR6b_1*02 | 360 | 12.2% | TAAR6b_1_AA*02 | 12.2% |
| 432F &T6-741R | Seg2 | TAAR6b_2*01 | 354 | 87.9% | TAAR6b_2_AA*01 | 87.9% |
| 432F &T6-741R | Seg2 | TAAR6b_2*02 | 354 | 83.3% | TAAR6b_2_AA*02 | 96.3% |
| 432F &T6-741R | Seg2 | TAAR6b_2*03 | 354 | 37% |  |  |
| 432F &T6-741R | Seg2 | TAAR6b_2*04 | 354 | 50.6% | TAAR6b_2_AA*03 | 50.6% |
| 432F &T6-741R | Seg2 | TAAR6b_2*05 | 354 | 1.1% | TAAR6b_2_AA*04 | 1.1% |
| **TAAR8a** | | | | | | |
| TAAR6-8F & TAAR6-8R | Seg1 | TAAR8a_1*01 | 393 | 99% | TAAR8a_1_AA*01 | 99.7% |
| TAAR6-8F & TAAR6-8R | Seg1 | TAAR8a_1*02 | 393 | 12.2% |  |  |
| TAAR6-8F & TAAR6-8R | Seg1 | TAAR8a_1*03 | 393 | 11.2% | TAAR8a_1_AA*02 | 11.2% |
| 478F & 802R | Seg2 | TAAR8a_2*01 | 394 | 100% | TAAR8a_2_AA*01 | 100% |
| 478F & 802R | Seg2 | TAAR8a_2*02 | 394 | 0.8% | TAAR8a_2_AA*02 | 0.8% |
| 478F & 802R | Seg2 | TAAR8a_2*03 | 394 | 0.5% | TAAR8a_2_AA*03 | 0.5% |
| **TAAR8b** | | | | | | |
| TAAR6-8F & TAAR6-8R | Seg1 | TAAR8b_1*01 | 393 | 95.7% | TAAR8b_1_AA*01 | 96.7% |
| TAAR6-8F & TAAR6-8R | Seg1 | TAAR8b_1*02 | 393 | 14% |  |  |
| TAAR6-8F & TAAR6-8R | Seg1 | TAAR8b_1*03 | 393 | 44% | TAAR8b_1_AA*02 | 44% |
| TAAR6-8F & TAAR6-8R | Seg1 | TAAR8b_1*04 | 393 | 13.7% | TAAR8b_1_AA*03 | 13.7% |
| TAAR6-8F & TAAR6-8R | Seg1 | TAAR8b_1*05 | 393 | 12.2% | TAAR8b_1_AA*04 | 12.2% |
| TAAR6-8F & TAAR6-8R | Seg1 | TAAR8b_1*07 | 393 | 1.5% |  |  |
| TAAR6-8F & TAAR6-8R | Seg1 | TAAR8b_1*06 | 393 | 1.8% | TAAR8b_1_AA*05 | 1.8% |
| **TAAR8c** | | | | | | |
| TAAR6-8F & TAAR6-8R | Seg1 | TAAR8c_1*01 | 393 | 99.5% | TAAR8c_1_AA*01 | 99.5% |
| TAAR6-8F & TAAR6-8R | Seg1 | TAAR8c_1*03 | 393 | 3.1% |  |  |
| TAAR6-8F & TAAR6-8R | Seg1 | TAAR8c_1*04 | 393 | 2.7% |  |  |
| TAAR6-8F & TAAR6-8R | Seg1 | TAAR8c_1*02 | 393 | 6.4% | TAAR8c_1_AA*02 | 6.4% |
| 478F & 802R | Seg2 | TAAR8c_2*01 | 395 | 99.5% | TAAR8c_2_AA*01 | 99.5% |
| 478F & 802R | Seg2 | TAAR8c_2*02 | 395 | 6.3% | TAAR8c_2_AA*02 | 6.3% |

| **Table S3**: Study population size and number of TAAR-typed meerkats per year. As numbers vary slightly by paralogue and segment, the count for each segment of each paralogue is provided, along with the overall number of TAAR-typed individuals. | | | | | | | | | | | |
| --- | --- | --- | --- | --- | --- | --- | --- | --- | --- | --- | --- |
| **Year** | **Study population size** | **TAAR-typed individuals** | 6a_1 | 6a_2 | 6b_1 | 6b_2 | 8a_1 | 8a_2 | 8b_1 | 8c_1 | 8c_2 |
| **1996** | **71** | **6** | 6 | 6 | 6 | 6 | 6 | 6 | 6 | 6 | 6 |
| **1997** | **163** | **13** | 13 | 12 | 12 | 12 | 13 | 13 | 13 | 13 | 13 |
| **1998** | **193** | **20** | 20 | 17 | 19 | 17 | 20 | 20 | 20 | 20 | 20 |
| **1999** | **296** | **35** | 35 | 22 | 34 | 22 | 35 | 35 | 35 | 35 | 35 |
| **2000** | **283** | **56** | 56 | 34 | 53 | 34 | 56 | 55 | 56 | 56 | 55 |
| **2001** | **364** | **62** | 62 | 36 | 60 | 36 | 62 | 61 | 62 | 62 | 61 |
| **2002** | **403** | **69** | 69 | 42 | 68 | 42 | 69 | 68 | 69 | 69 | 68 |
| **2003** | **372** | **64** | 63 | 42 | 62 | 42 | 63 | 64 | 63 | 63 | 64 |
| **2004** | **319** | **68** | 67 | 59 | 66 | 59 | 67 | 67 | 67 | 67 | 68 |
| **2005** | **388** | **90** | 86 | 81 | 80 | 81 | 86 | 89 | 86 | 86 | 90 |
| **2006** | **456** | **102** | 98 | 95 | 89 | 95 | 98 | 101 | 98 | 98 | 102 |
| **2007** | **388** | **97** | 94 | 91 | 85 | 91 | 94 | 96 | 94 | 94 | 97 |
| **2008** | **350** | **63** | 62 | 60 | 56 | 60 | 62 | 62 | 62 | 62 | 63 |
| **2009** | **433** | **70** | 70 | 68 | 61 | 68 | 70 | 69 | 70 | 70 | 70 |
| **2010** | **407** | **85** | 85 | 82 | 69 | 82 | 85 | 84 | 85 | 85 | 85 |
| **2011** | **518** | **101** | 101 | 98 | 86 | 98 | 101 | 101 | 101 | 101 | 101 |
| **2012** | **490** | **114** | 114 | 110 | 97 | 110 | 114 | 114 | 114 | 114 | 114 |
| **2013** | **378** | **108** | 108 | 105 | 91 | 105 | 108 | 108 | 108 | 108 | 108 |
| **2014** | **465** | **111** | 110 | 107 | 103 | 107 | 110 | 111 | 110 | 110 | 111 |
| **2015** | **461** | **108** | 107 | 104 | 103 | 104 | 107 | 107 | 107 | 107 | 107 |
| **2016** | **349** | **96** | 95 | 92 | 92 | 92 | 95 | 95 | 95 | 95 | 95 |
| **2017** | **283** | **62** | 62 | 60 | 60 | 60 | 62 | 60 | 62 | 62 | 60 |
| **2018** | **302** | **51** | 51 | 44 | 49 | 44 | 51 | 50 | 51 | 51 | 50 |
| **2019** | **275** | **42** | 42 | 34 | 38 | 34 | 42 | 41 | 42 | 42 | 41 |
| **2020** | **298** | **28** | 28 | 23 | 24 | 23 | 28 | 27 | 28 | 28 | 27 |
| **2021** | **167** | **21** | 21 | 16 | 18 | 16 | 21 | 21 | 21 | 21 | 21 |
| **2022** | **119** | **16** | 16 | 11 | 13 | 11 | 16 | 16 | 16 | 16 | 16 |
| **2023** | **60** | **8** | 8 | 6 | 7 | 6 | 8 | 8 | 8 | 8 | 8 |

| Table S4: Primers used to assess polymorphism in meerkat TAAR loci 1 to 9 by Sanger sequencing. | | | | | |
| --- | --- | --- | --- | --- | --- |
| Locus | Primer Pair | Primer Sequence | Product Size | Annealing Temp. | Samples |
| TAAR1 | TAAR1F1  TAAR1R2 | CCGTGCTTCCCTGTACAGTT  GGCCAGTACCCCAGATGTTT | 517 | 56°C | 10 |
| TAAR2 | TAAR2F3  TAAR2R1 | TCAAGCAGCTTCACACACCA  YCATCACTATTCCTAAAGTTTTGGC | 594 | 56°C | 10 |
| TAAR3 | TAAR3F3  TAAR3R3 | TATGCGACCACCATGACCAC  AGCTTTCCGAAACCATGGGT | 541 | 56°C | 10 |
| TAAR4 | TAAR4F4  TAAR4R3 | TGTGTGGTCATGCCCTTCAG  TGCTTAAAGTCTTGGTGGCCT | 541 | 56°C | 10 |
| TAAR5 | TAAR5F2  TAAR5R3 | TGTGGTGTTTGCTGTGTCCT  TAGGGTTGCAGGCTGAGTTG | 745 | 56°C | 10 |
| TAAR6 | TAAR6F1  TAAR6R2 | TGCAYTTCAAGCAGCTGCACT  TGCTACCACHGTGATACCCAGGG | 623 | 56°C | 10 |
| TAAR6 | 519F  1152R | TGATGATGGGCTGGAGGAGT  CCCCAAAACATCAATGAGTGCA | 591 | 57°C | 7 |
| TAAR6 | 198F  831R | CCTCCTGGTGGTGACTTCAA  ACCCAGGGTTTTAGCTGCTT | 593 | 57°C | 7 |
| TAAR7 | 220F  877R | TTTCTGATCGTGTCCCTGGC  TGACAAAACCCAGGAAGGCA | 617 | 58°C | 10 |
| TAAR8 | TAAR8F3  TAAR8R3 | TGCTTCATCTCCATCGACAGG  AAATCAAAGGGTTMATGGCTGA | 560 | 56°C | 10 |
| TAAR8 | 478F  802R | TTCACRGTGTCYGTGTCAGG  TCTTGGCYACTCKGG ATTTATAACT | 279 | 57°C | 10 |
| TAAR9 | TAAR9F2  TAAR9R1 | ACTTTCTGATCGCGTCCCTG  GCTTGGCTGAACTTTC | 510 | 56°C | 10 |

| Table S5: Primer pairs designed for Illumina sequencing of TAAR6 and TAA8 loci in meerkats. Several primer pairs were tested, but not used in the final analyses. Due to conserved sequences, one primer pair was sufficient to amplify segment 1 for all TAAR6 and TAAR8 loci. For segment 2, differences between TAAR6 and TAAR8 loci and variability between TAAR8a/c and TAAR8b required several specific primer pairs to target the particular paralogues. | | | | | |
| --- | --- | --- | --- | --- | --- |
| Locus | Primer Pair | Primer Sequence | Product Size [bp] | Annealing  Temp. | Included in final analyses |
| TAAR6/TAAR8-seg1 | TAAR6_8_NGS_F1  TAAR6_8_NGS_R1 | CTGGCTGTGTTTGGAAAC  CCAAAGTACCAGCAGCTCTC | 170 | 55°C | No |
| TAAR6/TAAR8-seg1 | TAAR6-8F  TAAR6-8R | CTTCAGCATGGTCAGGTCTGT  AACTCCTCCAKBCCATCATCA | 237 | 58°C | Yes |
| TAAR6/TAAR8-seg1 | TAAR6_8_NGS_F3TAAR6_8_NGS_R3 | CAGTGGTGSYSTGTTCTWCACRGGTG  TGATACCCAGKGTYTTMGCKGCTTTTC | 290 | 61°C | No |
| TAAR6-seg2 | 432F  T6-741R | CACGGTGTCTGTGTCAGGG  CCTGGCTTTGTTAGGCCCT | 271 | 58°C | Yes |
| TAAR8b-seg2 | 432F  T68b-741R | CACGGTGTCTGTGTCAGGG  CCTGGTTTTGTTAGGACAT | 271 | 58°C | Yes |
| TAAR8ac-seg2 | 478F  802R | TTCACRGTGTCYGTGTCAGG  TCTTGGCYACTCKGGATTTATAACT | 279 | 58°C | Yes |

| Table S6: Read coverage obtained from Illumina runs for each TAAR6 and TAAR8 segment, using the primer pairs detailed in Table S2. For quality-filtered reads, coverage is reported as the depth per sample and per paralogue. | | | | | | | |
| --- | --- | --- | --- | --- | --- | --- | --- |
| Primer-Pair | Read Type | Total | Mean | SD | Max | Min | Repeatability |
| All | Raw  Quality filtered | 18,732,276  14,243,831 | NA | NA | NA | NA | NA |
| TAAR6/TAAR8-seg1 | Raw  Quality filtered | 18,732,276  6,020,578 | 44,180  14,199 | 12,682  3,424 | 77,442  38,733 | 12,990  5,187 | 99.3% |
| TAAR6-seg2 | Raw  Quality filtered | 17,628,513  3,726,807 | 45,552  9,656 | 12,730  3,475 | 77,442  35,261 | 5,879  757 | 95.9% |
| TAAR8ac-seg2 | Raw  Quality filtered | 18,194,284  4,495,996 | 42,510  10,505 | 15,288  2,918 | 77,442  22,803 | 6,467  3,492 | 89.2% |
